# Functional Divergence in Solute Permeability between Ray-Finned Fish-Specific Paralogs of *aqp10*

**DOI:** 10.1093/gbe/evad221

**Published:** 2023-12-01

**Authors:** Genki Imaizumi, Kazutaka Ushio, Hidenori Nishihara, Ingo Braasch, Erika Watanabe, Shiori Kumagai, Tadaomi Furuta, Koji Matsuzaki, Michael F Romero, Akira Kato, Ayumi Nagashima

**Affiliations:** School of Life Science and Technology, Tokyo Institute of Technology, Yokohama, Japan; School of Life Science and Technology, Tokyo Institute of Technology, Yokohama, Japan; School of Life Science and Technology, Tokyo Institute of Technology, Yokohama, Japan; Department of Advanced Bioscience, Faculty of Agriculture, Kindai University, Nara, Japan; Department of Integrative Biology and Ecology, Evolution, and Behavior Program, College of Natural Science, Michigan State University, East Lansing, Michigan, USA; School of Life Science and Technology, Tokyo Institute of Technology, Yokohama, Japan; School of Life Science and Technology, Tokyo Institute of Technology, Yokohama, Japan; School of Life Science and Technology, Tokyo Institute of Technology, Yokohama, Japan; Marine Science Museum, Fukushima Prefecture (Aquamarine Fukushima, AMF), Iwaki, Japan; Department of Physiology and Biomedical Engineering, Mayo Clinic College of Medicine & Science, Rochester, Minnesota, USA; Department of Nephrology and Hypertension, Mayo Clinic College of Medicine & Science, Rochester, Minnesota, USA; School of Life Science and Technology, Tokyo Institute of Technology, Yokohama, Japan; School of Life Science and Technology, Tokyo Institute of Technology, Yokohama, Japan

**Keywords:** aquaporin 10, aquaglyceroporin, paralog, ray-finned fish, subfunctionalization, neofunctionalization

## Abstract

Aquaporin (Aqp) 10 is a member of the aquaglyceroporin subfamily of water channels, and human Aqp10 is permeable to solutes such as glycerol, urea, and boric acid. Tetrapods have a single *aqp10* gene, whereas ray-finned fishes have paralogs of this gene through tandem duplication, whole-genome duplication, and subsequent deletion. A previous study on Aqps in the Japanese pufferfish *Takifugu rubripes* showed that one pufferfish paralog, Aqp10.2b, was permeable to water and glycerol, but not to urea and boric acid. To understand the functional differences of Aqp10s between humans and pufferfish from an evolutionary perspective, we analyzed Aqp10s from an amphibian (*Xenopus laevis*) and a lobe-finned fish (*Protopterus annectens*) and Aqp10.1 and Aqp10.2 from several ray-finned fishes (*Polypterus senegalus*, *Lepisosteus oculatus*, *Danio rerio*, and *Clupea pallasii*). The expression of tetrapod and lobe-finned fish Aqp10s and Aqp10.1-derived Aqps in ray-finned fishes in *Xenopus* oocytes increased the membrane permeabilities to water, glycerol, urea, and boric acid. In contrast, Aqp10.2-derived Aqps in ray-finned fishes increased water and glycerol permeabilities, whereas those of urea and boric acid were much weaker than those of Aqp10.1-derived Aqps. These results indicate that water, glycerol, urea, and boric acid permeabilities are plesiomorphic activities of Aqp10s and that the ray-finned fish-specific Aqp10.2 paralogs have secondarily reduced or lost urea and boric acid permeability.

**Significance**

Aquaporin (Aqp) 10 is a member of the aquaglyceroporin subfamily transporting small, uncharged solutes in addition to water. Differences in the solute permeabilities of Aqp10s between tetrapods and fishes have been identified, but it remains unclear when and how these differences arose. Water, glycerol, urea, and boric acid permeability was suggested as a plesiomorphic activity of Aqp10s and was conserved in Aqp10s of a tetrapod, a lobe-finned fish, and the Aqp10.1 paralogs in ray-finned fishes. On the other hand, the permeabilities of the Aqp10.2 paralogs to urea and boric acid were much weaker than those of plesiomorphic Aqp10s. This difference in activity between the specific Aqp10 paralogs of ray-finned fish suggests functional divergence following their tandem gene duplication.

## Introduction

Aquaporins (Aqps) are a family of water channel proteins that contain six-transmembrane domains (Borgnia et al. 1999; Agre et al. 2002; Azad et al. 2021) (Note that, in this article, protein names of all species are shown with the first letter capitalized rather than the full name italicized and gene names of all species are shown as lowercase and italicized). Most mammals, including human, possess 13 members of the aquaporin family (Aqp0–12), of which Aqp3, 7, 9, and 10 transport small, uncharged solutes, such as glycerol and urea, in addition to water. These four proteins are grouped into the aquaglyceroporin subfamily. The *aqp10* gene of certain rodents (e.g., mice) and ruminants (e.g., cows, sheep, and goats) has been lost or transitioned to a pseudogene (Morinaga et al. 2002; Tanaka et al. 2015). Studies on the Aqp8s of humans and fishes indicate that this protein is also permeable to water and small uncharged molecules such as urea, but not to glycerol; therefore, Aqp8 is categorized as a member of the water and urea channel subfamily (Tingaud-Sequeira et al. 2010; Ushio et al. 2022; Kumagai et al. 2023). In contrast, Aqp1, 2, 4, and 5 are permeable to water but not to glycerol, urea, or other compounds, and these Aqps are categorized as classical or water-selective.

Aquaglyceroporins in mammals are involved in physiological processes such as gastrointestinal functioning, hepatic and adipocyte metabolism, skin elasticity, and pancreatic β-cell regulation (Lebeck 2014; Laforenza et al. 2016; Calamita and Delporte 2021). Their expression differs by location: Aqp7 and 10 in the apical membrane of enterocytes, Aqp3 in the basolateral membrane of enterocytes, Aqp9 in the plasma membrane of hepatocytes, and Aqp3, 7, 9, and 10 in adipose tissue. These aquaglyceroporins play significant roles in glycerol metabolism. In addition, Aqp3 is expressed in various tissues, including epidermal keratinocytes. In Aqp3-deficient mice, the epidermis has reduced water and glycerol content in the stratum corneum, reduced skin elasticity, impaired epidermal biosynthesis, and delayed wound healing (Hara-Chikuma and Verkman 2008). Aqp7 is also expressed in pancreatic β-cells, and Aqp7-mediated glycerol uptake is involved in β-cell insulin secretion (Matsumura et al. 2007).

**Fig. 1.**
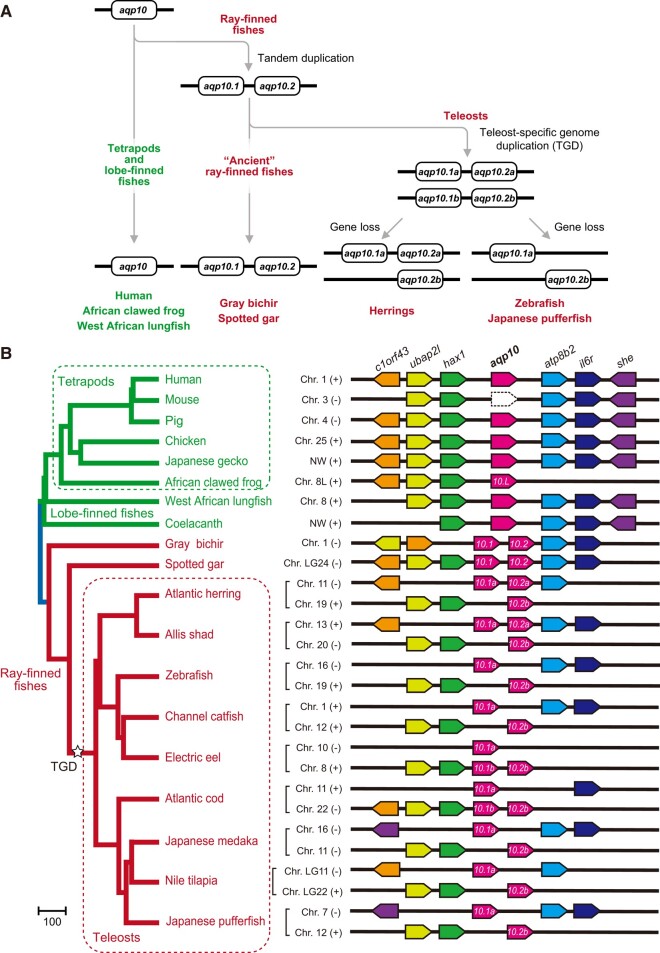
Evolutionary relationship of *aqp10* genes in the bony vertebrates analyzed in this study. (*A*) Flowchart showing how ray-finned fishes developed more than one *aqp10* gene through tandem gene duplication, TGD, and deletion (Yilmaz et al. 2020). (*B*) Synteny analyses of *aqp10* genes in bony vertebrates. (+) and (−) represent the right and left orientations, respectively, of the genome sequences in the NCBI and ENSEMBL databases. Synteny analysis was performed using the Ensembl genome browser (Martin et al. 2023) and NCBI genome viewer (Rangwala et al. 2021) with genome databases of various species (supplementary table S3, Supplementary Material online). Arrow-shaped boxes indicate the orientation of each gene. Dotted arrow-shaped boxes indicate pseudogenes. The phylogeny of bony vertebrate species based on the TimeTree database (http://www.timetree.org/) (Kumar et al. 2017) is shown on the left.

Detailed analyses of the Aqp superfamily in vertebrates (Finn et al. 2014; Finn and Cerda 2015; Chauvigne et al. 2019; Yilmaz et al. 2020) revealed 17 subfamilies (Aqp0–16), of which Aqp3, 7, 9, 10, and 13 belong to the subfamily aquaglyceroporins. Aqp3, 7, 9, and 10 are present in a relatively large number of species, whereas Aqp13 is present only in prototherian mammals and in amphibians (Finn et al. 2014; Finn and Cerda 2015; Chauvigne et al. 2019; Yilmaz et al. 2020). Teleost fishes have a high number of *aqp3*, *aqp7*, *aqp9*, and *aqp10* genes due to lineage- and species-specific gene duplications, whole-genome duplications, and deletions; their evolutionary history was described in detail by Yilmaz et al. (Yilmaz et al. 2020). The ancestral ray-finned fish acquired *aqp10.1* and *aqp10.2* through tandem duplication of *aqp10* (fig. 1*A*) and lost the *aqp13* gene. The former names of *aqp10.1* and *aqp10.2* are *aqp10a* (or *aqp10aa*) and *aqp10b* (or *aqp10ab*), respectively. In this article, gene symbols of tandem duplicates are appended with “.1” or “.2”, whereas those of ohnologs, gene duplicates originating from genome duplication, are indicated by “a” or “b” following the Zebrafish Nomenclature Conventions (http://zfin.org/). Consequently, ancestral ray-finned fishes could have possessed five aquaglyceroporin genes: *aqp3*, *aqp7*, *aqp9*, *aqp10.1*, and *aqp10.2* (Yilmaz et al. 2020). In teleost fishes, these genes were doubled during the teleost-specific genome duplication (TGD), followed by lineage-specific gene losses or duplications (Yilmaz et al. 2020). Therefore, the number of paralogs, including ohnologs of aquaglyceroporin genes in teleosts, varies among species and lineages (Yilmaz et al. 2020). The majority of teleosts has one or two *aqp3* genes (*aqp3a* and *aqp3b*), whereas salmonids have a greater number of paralogs due to their lineage-specific tandem duplication (*aqp3a1a* and *aqp3a1b*) and whole-genome duplication (Yilmaz et al. 2020). Teleosts with a single *aqp3* gene usually retain *aqp3a* as opposed to *aqp3b*. Most teleosts possess one *aqp7* gene and two *aqp9* genes (*aqp9a* and *aqp9b*). The TGD resulted in *aqp10.1a* and *aqp10.1b* generated from the ancestral *aqp10.1* and *aqp10.2a* and *aqp10.2b* from the ancestral *aqp10.2* (fig. 1*A*). Note that the former names of *aqp10.1a*, *aqp10.1b*, *aqp10.2a*, and *aqp10.2b* are *aqp10aa*, *aqp10ba*, *aqp10ab*, and *aqp10bb*, respectively. After reciprocal genes losses from TGD-duplicated chromosomes, *aqp10.1a* and *aqp10.2b* genes are often retained on separate chromosomes, and most teleost species possess both *aqp10.1* and *aqp10.2*. *Polypterus* and *Lepisosteus*, so-called ancient ray-finned fishes, retained tandemly located *aqp10.1* and *aqp10.2* on the same chromosome, which suggest that ray-finned fishes require both *aqp10.1* and *aqp10.2*. Some teleost lineages, such as cod, have multiple *aqp10.1* genes (*aqp10.1a* and *aqp10.1b*), whereas others, such as herring, have multiple *aqp10.2* types (*aqp10.2a* and *aqp10.2b*) (fig. 1*B* and supplementary fig. S1, Supplementary Material online).

Recently, human (*Homo sapiens*) and Japanese pufferfish (*Takifugu rubripes*) Aqps (HsaAqps and TruAqps, respectively) were expressed in *Xenopus* oocytes and their water, glycerol, urea, and boric acid permeabilities were analyzed (Ushio et al. 2022; Kumagai et al. 2023). The solute selectivity of the Aqp orthologs between humans and Japanese pufferfishes was similar, except for that of Aqp10; oocytes expressing HsaAqp10 were permeable to water, glycerol, urea, and boric acid, whereas those expressing TruAqp10.2b were permeable to water and glycerol but not to urea and boric acid. This difference in activity may have occurred after the two species diverged. These evolutionary adaptions in solute selectivity could present a promising model for analyzing the evolution of solute permeability in aquaglyceroporins. The plesiomorphic activities of Aqp10 and the evolutionary timing of changes in solute selectivity change during the evolution of bony vertebrates are unclear.

In this study, to elucidate the evolutionary history of solute selectivity of Aqp10, we analyzed and compared the permeability and evolutionary relationships of Aqp10s in eight bony vertebrate species: human, African clawed frog, West African lungfish, gray bichir, spotted gar, zebrafish, Pacific herring, and Japanese pufferfish. The results indicated that water, glycerol, urea, and boric acid permeabilities were plesiomorphic activities of Aqp10 and the ray-finned fish-specific paralog Aqp10.2 either lost or weakened urea and boric acid permeability from its common ancestor, that is to say, they may be novel examples of Aqp functional divergence by subfunction losses by one of the two duplicates.

## Results

### Evolutional Relationships of the *aqp10* Genes in the Vertebrate Species

The evolutionary relationships of the *aqp10* paralogs used in the present study are summarized in fig. 1*A* according to the results of Yilmaz et al. (Yilmaz et al. 2020). We confirmed the evolutional relationship using phylogenetic and conserved synteny analyses (fig. 1*B*, supplementary fig. S1, Supplementary Material online) and that 1) tetrapods and lobe-finned fishes had a single *aqp10*; 2) a tandem gene duplication in ancestral ray-finned fishes (i.e., the common ancestor of *Polypterus*, *Lepisosteus*, and teleosts) generated *aqp10.1* and *aqp10.2*; 3) the TGD generated *aqp10.1a* and *aqp10.1b* from *aqp10.1* and *aqp10.2a* and *aqp10.2b* from *aqp10.2*, respectively, in ancestral teleost species; and 4) herring lost the *aqp10.1b* gene, whereas the Atlantic cod and electric eel lost *aqp10.2a* and the zebrafish and Japanese pufferfish lost both *aqp10.1b* and *aqp10.2a*.

### Tissue Distribution of *aqp10*s in the African Clawed Frog and Spotted Gar

**Fig. 2.**
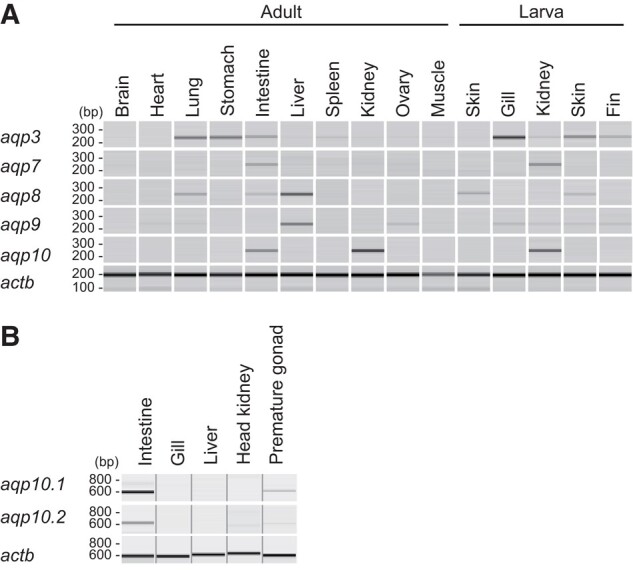
Tissue distribution of *aqp10s* in the African clawed frog (*A*) and spotted gar (*B*). (*A*) Expression profiles of *aqp10* and related aquaglyceroporin genes in African clawed frog tissues were determined using semiquantitative RT-PCR. Pseudo-gel images of the PCR products were generated using a microchip electrophoresis system. *actb* (β-actin gene) were used as an internal control. (*B*) Expression profiles of *aqp10.1* and *aqp10.2* in spotted gar tissues were determined using semiquantitative RT-PCR. *actb* was used as an internal control gene.

The expression distributions of *aqp10* in African clawed frog tissue and *aqp10.1* and aqp*10.2* in spotted gar were analyzed by semiquantitative reverse transcription polymerase chain reaction (RT-PCR). In addition, the tissue distributions of *aqp3*, *aqp7*, *aqp8*, and *aqp9* were investigated in the African clawed frog (fig. 2*A*). The expressions were as follows: *aqp10* was found in the intestines and kidneys primarily; *aqp3* in the lungs, stomach, intestine, liver, spleen, kidney, ovaries, skeletal muscle, skin, gills, and fins; *aqp7* in the intestine and larval kidneys; *aqp8* in the lungs, intestine, liver, and skin; and *aqp9* in the heart, lungs, liver, ovaries, skeletal muscles, gills, larval kidneys, larval skin, and fins. In spotted gar tissues, *aqp10.1* and *10.2* were highly expressed in the intestine (fig. 2*B*).

### Solute and Water Permeability of Aqp10s in Tetrapods and a Lobe-Finned Fish

**Fig. 3.**
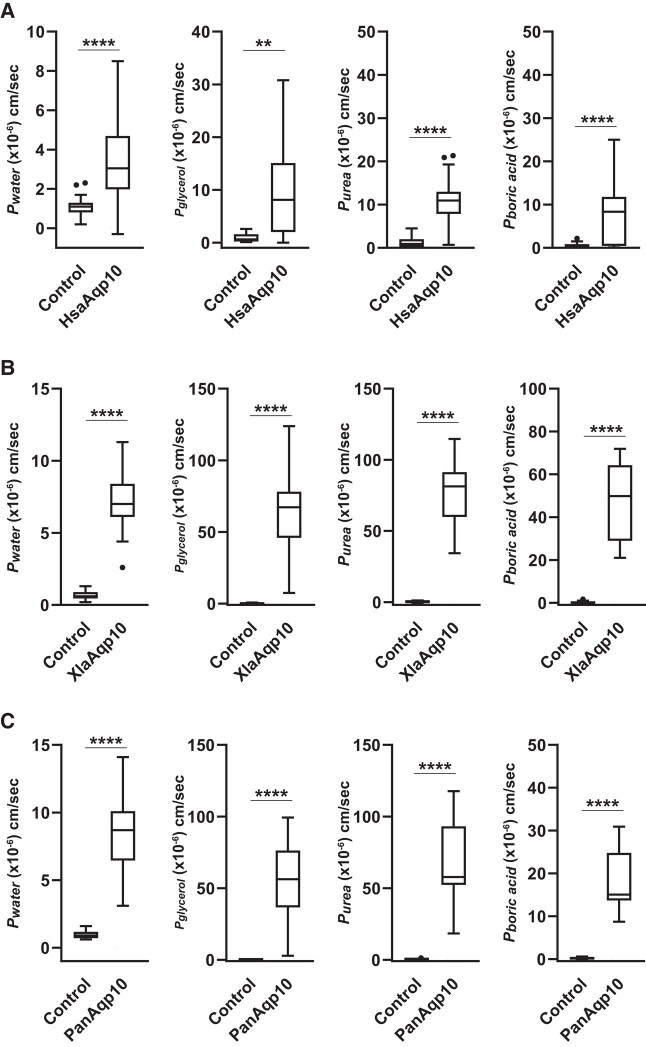
Water and solute (glycerol, urea, and boric acid) permeabilities of Aqp10*s* in humans (HsaAqp10) (*A*), African clawed frogs (XlaAqp10) (*B*), and West African lungfishes (PanAqp10) (*C*) as measured by a swelling assay. The change in the volume of oocytes expressing each Aqp10 was compared with that of control oocytes. Values are presented as interquartile ranges from the 25 to 75 percentile (box), range (whiskers), outliers (>1.5× the interquartile range above the upper quartile), and median (line in the box). Mean values, standard deviations, and total numbers of assayed oocytes are summarized in table 1. Statistical significance was evaluated by an unpaired *t*-test (****P < 0.0001; **P < 0.01).

cRNAs for the Aqp10s of the African clawed frog (XlaAqp10) and West African lungfish (PanAqp10) were injected into *Xenopus* oocytes, and their solute and water permeabilities were analyzed using a swelling assay. For comparison, we also analyzed human Aqp10 (HsaAqp10). Oocytes expressing HsaAqp10, XlaAqp10, and PanAqp10 showed significant volume gains and increases in *P*_water_ in the hypo-osmotic solution (fig. 3, table 1), suggesting that these Aqp10s act as water channels in the plasma membranes of oocytes.

**Table 1:** Water and Solute Permeability Measurements of Aqp10s in Oocytes

Protein	*P*_water_ (×10^−6^ cm/s, 100 mosM Inside Osmotic Gradient)	*P*_glycerol_ (×10^−6^ cm/s, 180 mM Outside Solute Gradient)	*P*_urea_ (×10^−6^ cm/s, 180 mM Outside Solute Gradient)	*P*_boric acid_ (×10^−6^ cm/s, 180 mM Outside Solute Gradient)
HsaAqp10	3.3 ± 2.2 (30)	9.7 ± 8.7 (15)	10.7 ± 5.5 (22)	7.6 ± 6.6 (22)
Control1	1.1 ± 0.5 (27)	0.9 ± 0.9 (11)	1.1 ± 1.1 (24)	0.6 ± 0.5 (24)
XlaAqp10	7.4 ± 2.3 (15)	62.0 ± 27.7 (15)	78.5 ± 23.6 (15)	47.8 ± 17.5 (12)
Control2	0.7 ± 0.3 (13)	−0.1 ± 0.5 (13)	0.2 ± 0.7 (14)	0.2 ± 0.7 (11)
PanAqp10	8.2 ± 2.9 (16)	55.2 ± 27.4 (18)	68.1 ± 28.9 (18)	18.0 ± 6.7 (16)
Control3	1.0 ± 0.3 (16)	0.0 ± 0.3 (16)	0.0 ± 0.4 (15)	0.2 ± 0.3 (20)
TruAqp10.2b	5.7 ± 2.9 (10)	35.3 ± 21.1 (9)	2.1 ± 1.2 (10)	1.7 ± 0.6 (9)
Control4	1.5 ± 0.4 (10)	0.6 ± 1.1 (10)	1.1 ± 1.6 (10)	0.8 ± 0.7 (10)
DreAqp10.1a	12.3 ± 5.6 (9)	45.9 ± 26.6 (17)	79.7 ± 28.7 (11)	48.0 ± 23.6 (5)
DreAqp10.2b	6.6 ± 4.4 (9)	102.1 ± 25.2 (10)	12.0 ± 4.1 (14)	6.1 ± 3 (7)
Control5	0.8 ± 0.2 (9)	0.1 ± 0.4 (14)	0.2 ± 0.2 (14)	0.5 ± 0.4 (9)
CpaAqp10.1a	7.3 ± 2.8 (11)	72.7 ± 25.1 (6)	107 ± 41.1 (9)	70.9 ± 26.5 (13)
CpaAqp10.2a	5.9 ± 2.6 (10)	34.3 ± 13.6 (11)	31.5 ± 10.7 (7)	30.9 ± 15.8 (12)
CpaAqp10.2b	8.2 ± 5.6 (12)	131.2 ± 19 (4)	26.4 ± 3.8 (10)	16.0 ± 3.1 (11)
Control6	0.9 ± 0.3 (11)	0.0 ± 0.3 (11)	0.0 ± 0.4 (19)	0.2 ± 0.3 (24)
PseAqp10.1	10.0 ± 2.3 (10)	73.2 ± 32.8 (9)	85 ± 25.4 (7)	61.1 ± 27.8 (7)
PseAqp10.2	9.4 ± 4.6 (12)	79.6 ± 17.9 (7)	4.0 ± 0.7 (8)	2.8 ± 1.2 (7)
Control7	0.9 ± 0.3 (11)	0.0 ± 0.3 (11)	0.1 ± 0.5 (11)	0.1 ± 0.2 (16)
LocAqp10.1	7.2 ± 2.5 (11)	61.8 ± 25.9 (8)	104 ± 36 (10)	66.9 ± 28.4 (9)
LocAqp10.2	6.3 ± 2.2 (37)	46.1 ± 18.3 (40)	8.8 ± 3.3 (32)	4.1 ± 2 (37)
Control8	0.9 ± 0.3 (37)	0.2 ± 0.9 (35)	0.0 ± 0.4 (34)	0.2 ± 0.5 (36)

Values are presented as means ± standard deviations. Numbers in parentheses indicate the total number of oocytes assayed.

The glycerol, urea, and boric acid permeabilities of oocytes expressing HsaAqp10, XlaAqp10, and PanAqp10 were analyzed by a swelling assay using an iso-osmotic solution containing 180 mM of glycerol, urea, or boric acid, respectively. Oocytes expressing HsaAqp10, XlaAqp10, and PanAqp10 showed significant volume gains and increases in *P*_glycerol_, *P*_urea_, and *P*_boric acid_ (fig. 3, table 1), suggesting that these Aqp10s act as channels for all three compounds.

### Solute and Water Permeability of Aqp10s of Teleost Species That Possess Two *aqp10* Paralogs, *aqp10.1a* and *aqp10.2b*

**Fig. 4.**
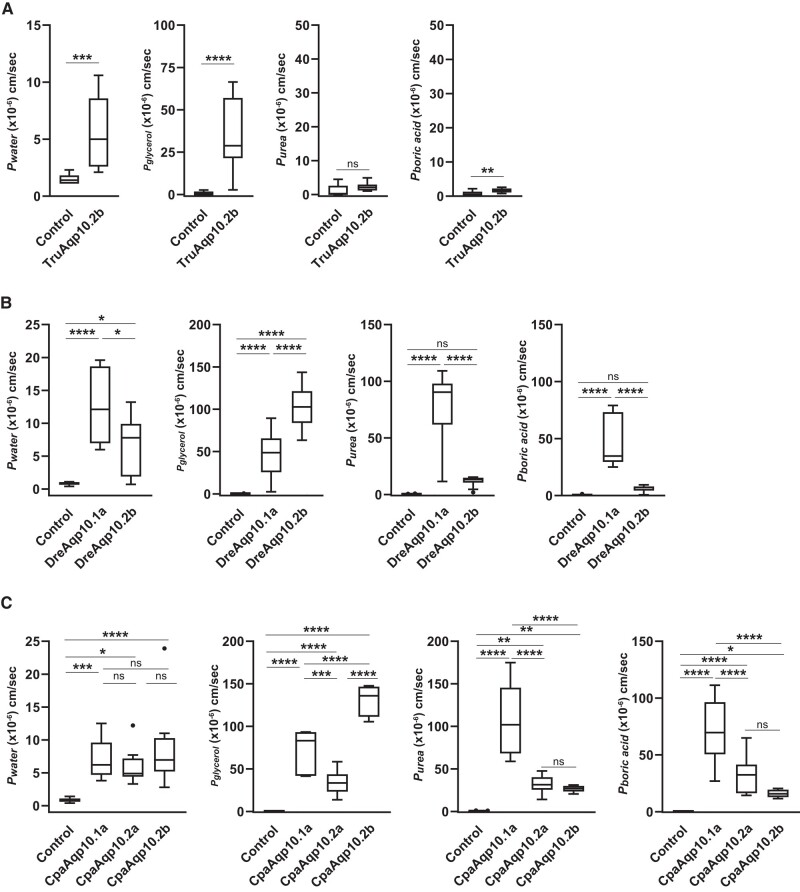
Water and solute (glycerol, urea, and boric acid) permeabilities of Aqp10*s* in Japanese pufferfishes (TruAqp10.2*b*) (*A*), zebrafishes (DreAqp10*s*) (*B*), and pacific herring (CpaAqp10*s*) (*C*) as measured by a swelling assay. The change in the volume of oocytes expressing each Aqp10 was compared with that of control oocytes. Values are presented as interquartile ranges from the 25 to75 percentiles (box), range (whiskers), outliers (>1.5× the interquartile range above the upper quartile), and median (line in the box). Mean values, standard deviations, and total numbers of assayed oocytes are summarized in table 1. Statistical significance for TruAqp10.2*b* was evaluated by an unpaired *t*-test (****P < 0.0001; ***P < 0.001). Statistical significance for DreAqp10*s* and CpaAqp10*s* was assessed by an ANOVA followed by Tukey’s test (****P < 0.0001; ***P <0.001; **P < 0.01; *P < 0.05).

Zebrafishes and Japanese pufferfishes possess two *aqp10* paralogs, *aqp10.1a* and *aqp10.2b* (fig. 1), which is typical for teleosts (Yilmaz et al. 2020). The water permeabilities of zebrafish oocytes expressing Aqp10s (DreAqp10.1a and 10.2b) were analyzed using a swelling assay. In addition, we analyzed Japanese pufferfish Aqp10.2b (TruAqp10.2b) for comparison. We did not analyze TruAqp10.1a in this study because, in a previous study, TruAqp10.1a did not show activity when expressed in *Xenopus* oocytes for unknown reasons (Kumagai et al. 2023). Oocytes expressing Aqp10s showed significant volume gains and increases in *P*_water_ in the hypo-osmotic solution (fig. 4*A* and *B*, table 1), suggesting that these Aqp10s act as water channels in the plasma membrane of oocytes.

The glycerol, urea, and boric acid permeabilities of oocytes expressing TruAqp10.2b, DreAqp10.1a, and DreAqp10.2b were similarly analyzed using a swelling assay with an iso-osmotic solution containing the solutes. In the iso-osmotic solution containing glycerol, oocytes expressing TruAqp10.2b, DreAqp10.1a, and DreAqp10.2b showed significant volume gains and increases in *P*_glycerol_ (fig. 4*A* and *B*, table 1), suggesting that these Aqp10s act as glycerol channels. In these experiments, the average *P*_glycerol_ value for DreAqp10.2b was 2.2 times higher than that of DreAqp10.1a, whereas the average *P*_water_ value for DreAqp10.2b was 1.9 times lower than that of DreAqp10.1a.

In the iso-osmotic solution containing urea or boric acid, DreAqp10.1a, but not DreAqp10.2b, showed significant volume gains and increases in *P*_urea_ and *P*_boric acid_ (fig. 4*B*, table 1), suggesting that DreAqp10.1a, but not DreAqp10.2b, acts as a urea and boric acid channel. TruAqp10.2b showed a slight increase in *P*_boric acid_ but no increase in *P*_urea_ (fig. 4*A*, table 1).

### Solute and Water Permeability of Aqp10s of Teleost Species That Possess Three *aqp10* Paralogs, One *aqp10.1* and Two *aqp10.2* Ohnologs

Herring (Martinez Barrio et al. 2016) and shad (Sabatino et al. 2022) are unique in that they harbor one *aqp10.1* (*aqp10.1a*) and two *aqp10.2 *s (*aqp10.2a* and *aqp10.2b*) (fig. 1) (Yilmaz et al. 2020); therefore, to understand the activity of Aqp10.2 ohnologs in teleosts, solute and water permeabilities of Aqp10s of Pacific herring (CpaAqp10.1a, 10.2a, and 10.2b) were analyzed by the *Xenopus* oocyte swelling assay. Oocytes expressing CpaAqp10s showed significant volume gains and increases in *P*_water_ in the hypo-osmotic solution (fig. 4*C*, table 1), suggesting that these Aqp10s act as water channels in the plasma membranes of oocytes.

In the iso-osmotic solution containing glycerol, oocytes expressing CpaAqp10s showed significant volume gains and increases in *P*_glycerol_ (fig. 4*C*, table 1), suggesting that these Aqp10s act as glycerol channels. In these experiments, the average values of *P*_glycerol_ for CpaAqp10.1a and 10.2b were 2.1 and 3.8 times higher than those of CpaAqp10.2a, respectively, whereas the averages of *P*_water_ were not significantly different among the CpaAqp10s.

In the iso-osmotic solution containing urea and boric acid, oocytes expressing CpaAqp10.1a showed significant cell volume gains and increases in *P*_urea_ and *P*_boric acid_ (fig. 4*C*, table 1), suggesting that CpaAqp10.1a acts as a urea and boric acid channel. CpaAqp10.2a and 10.2b showed a slight increase in *P*_urea_ and *P*_boric acid_ (fig. 4*C*, table 1). However, in these experiments, the average values of *P*_urea_ and *P*_boric acid_ of CpaAqp10.2a and 10.2b were 4.4–2.3 times lower than those of CpaAqp10.1a.

### Solute and Water Permeability of *Xenopus* Oocytes Expressing Aqp10s of “Ancient” Ray-Finned Fishes

**Fig. 5.**
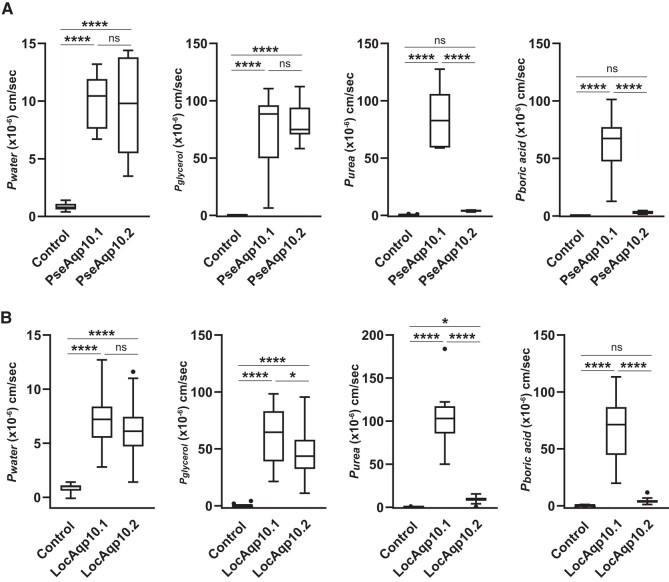
Water and solute (glycerol, urea, and boric acid) permeabilities of Aqp10*s* in gray bichirs (PseAqp10*s*) (*A*) and spotted gars (LocAqp10*s*) (*B*) as measured by a swelling assay. The change in the volume of oocytes expressing each Aqp10 was compared with that of control oocytes. Values are presented as interquartile ranges from the 25 to 75 percentiles (box), range (whiskers), outliers (>1.5× the interquartile range above the upper quartile), and median (line in the box). Mean values, standard deviations, and total numbers of assayed oocytes are summarized in table 1. Statistical significance was evaluated by an unpaired *t*-test (****P < 0.0001; *P < 0.05).

Gray bichir and spotted gar possess tandemly located *aqp10.1* and *aqp10.2* genes (fig. 1) (Yilmaz et al. 2020), and the Aqp10.1 and 10.2 of these species (PseAqp10s and LocAqp10s, respectively) were analyzed using a swelling assay. Oocytes expressing PseAqp10.1, PseAqp10.2, LocAqp10.1, and LocAqp10.2 showed significant volume gains and increases in *P*_water_ in the hypo-osmotic solution (fig. 5, table 1), suggesting that these Aqp10s act as water channels in the plasma membranes of oocytes.

The glycerol, urea, and boric acid permeabilities of oocytes expressing PseAqp10.1, PseAqp10.2, LocAqp10.1, and LocAqp10.2 were similarly analyzed by a swelling assay using an iso-osmotic solution containing solutes. Oocytes expressing PseAqp10s and LocAqp10s in the iso-osmotic solution containing glycerol showed significant volume gains and increases in *P*_glycerol_ (fig. 5, table 1), suggesting that these Aqp10s act as glycerol channels.

For the iso-osmotic solution containing urea or boric acid, PseAqp10.1 and LocAqp10.1, but not PseAqp10.2 and LocAqp10.2, showed significant volume gains and increases in *P*_urea_ and *P*_boric acid_ (fig. 5, table 1), suggesting that PseAqp10.1 and LocAqp10.1, but not PseAqp10.2 and LocAqp10.2, act as urea and boric acid channels.

## Discussion

The present study showed that Aqp10.2, which is an Aqp10 paralog of ray-finned fishes, has little or no urea nor boric acid transport activity and that these activities were systematically reduced and lost through evolution in the common ancestor of these fishes. According to Yilmaz et al. (Yilmaz et al. 2020), Aqp10 is widely expressed in cartilaginous fishes and bony vertebrates. Among the bony vertebrates, tetrapods and lobe-finned fishes have a single *aqp10*, whereas ray-finned fishes have two or more *aqp10* paralogs. Ancestors of ray-finned fishes acquired *aqp10.1* and *aqp10.2* through tandem duplication, and most extant species possess one or more genes derived from each of *aqp10.1* and *aqp10.2*. A previous study showed that human Aqp10 is permeable to water, glycerol, urea, and boric acid, whereas Japanese pufferfish Aqp10.2b, which is a paralog (e.g., ohnolog) of Aqp10.2, is permeable to water and glycerol, but not to urea or boric acid. In this study, we clarified the timing and history by which Aqp10 solute permeability evolved by analyzing the activity of 13 Aqp10s from eight bony vertebrate species.

The analysis of Aqp10 activity in an amphibian (the African clawed frog) and lobe-finned fish (the West African lungfish) showed permeability to water, glycerol, urea, and boric acid similar to that of human Aqp10, suggesting that solute selectivity is a common property of this protein in tetrapods and lobe-finned fishes (fig. 3). A subsequent analysis of Aqp10 activity of two zebrafish paralogs, Aqp10.1a and 10.2b, showed that Aqp10.1a was permeable to water, glycerol, urea, and boric acid, similar to human Aqp10. In contrast, Aqp10.2b was permeable to water and glycerol, but not to urea and boric acid, similar to the Japanese pufferfish Aqp10.2b (fig. 4*A* and *B*). This result indicates that urea and boric acid impermeability is a characteristic property of Aqp10.2b and is conserved among teleost species.

To determine when the Aqp10 solute selectivity changes occurred, we analyzed the Aqp10.1 and 10.2 functions in the “ancient” ray-finned fishes gray bichir and spotted gar (fig. 5). We found that Aqp10.1 was permeable to water, glycerol, urea, and boric acid in both species, similar to the Aqp10.1a in teleosts and the Aqp10s of tetrapods and lobe-finned fishes. Aqp10.2, however, was permeable to water and glycerol, but not to urea and boric acid, similar to Aqp10.2b in teleosts. These results strongly suggest that water, glycerol, urea, and boric acid permeabilities are plesiomorphic activities of Aqp10 that were retained by the Aqp10.1 of ray-finned fishes. In contrast, Aqp10.2 lost or weakened urea and boric acid permeabilities during evolution of the common ancestral species of ray-finned fishes.

**Fig. 6.**
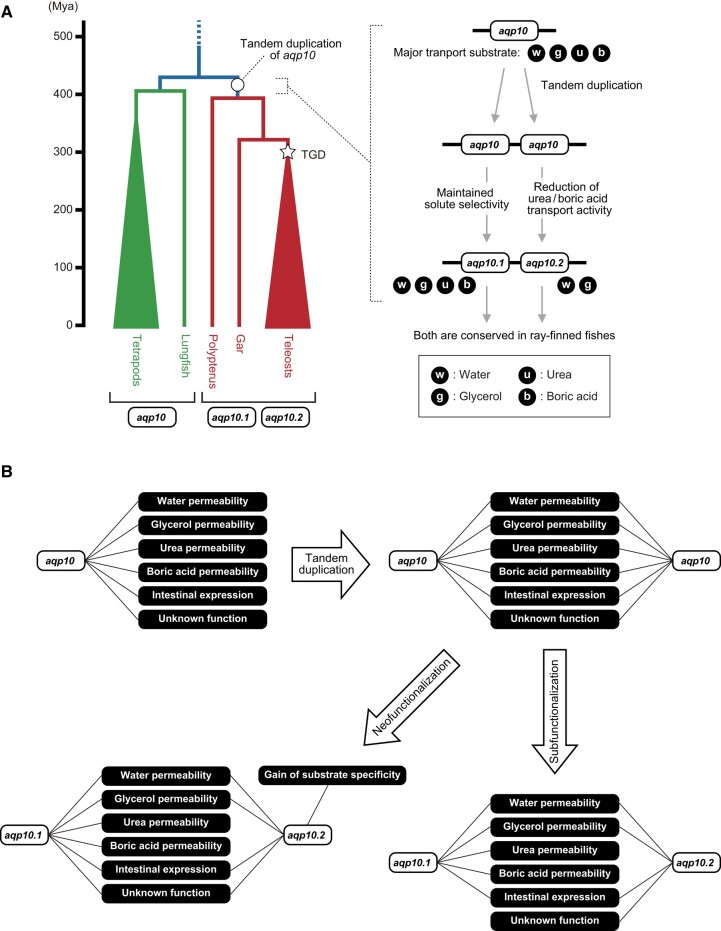
Evolutionary model and timing of *aqp10.1* and *aqp10.2* in ray-finned fishes. (*A*) The phylogeny of bony vertebrate species and time scale generated based on the TimeTree database (http://www.timetree.org/) (Kumar et al. 2017) is shown on the left. The right panel shows the hypothetical history of the method by which Aqp10.2 reduced or lost its urea and boric acid transport activity during evolution. TGD, teleost-specific genome duplication. (*B*) Evolutionary models of the functional divergence were illustrated by the model presented by He and Zhang (He and Zhang 2005).

To confirm whether the characteristics of Aqp10.2 were retained in both teleost-specific Aqp10 ohnologs, we used herring for our evaluation because these fishes retain *aqp10.2a* and *aqp10.2b*, whereas many teleosts only possess *aqp10.2b* (Yilmaz et al. 2020). The results indicated that CpaAqp10.1a increased *P*_water_, *P*_glycerol_, *P*_urea_, and *P*_boric acid_, whereas CpaAqp10.2a and 10.2b increased *P*_water_ and *P*_glycerol_, and *P*_urea_ and *P*_boric acid_ were much weaker than those of CpaAqp10.1a (fig. 4*C*). This result confirmed that Aqp10.2 in ancestral ray-finned species already had an absent or weakened urea and boric acid transport activity, and these characteristics were retained in teleost ohnologs. The presumed history of this lost or weakened urea and boric acid transport activity of teleost Aqp10.2 is illustrated in fig. 6.

Ray-finned fishes have both Aqp10.1 and 10.2; however, the physiological roles and reasons for retaining two types of Aqp10 with different solute permeabilities are not clear. So far, no reports have been identified that examine Aqp10 protein tissue localization in ray-finned fishes using immunohistochemistry, leaving the physiological functions of fish Aqp10s unclear, although the tissue distribution of Aqp10 in ray-finned fishes at the mRNA level has been reported in the zebrafish (Tingaud-Sequeira et al. 2010), Japanese pufferfish (Kumagai et al. 2023), Japanese eel (Kim et al. 2010), yellow croaker (Liu et al. 2019), and Atlantic salmon (Yilmaz et al. 2020). In the present study, we analyzed the distribution of Aqp10 mRNA in the spotted gar and found that both Aqp10.1 and Aqp10.2 were highly expressed in the intestine. Further analyses would be required to understand the roles of Aqp10.1 and 10.2 in ray-finned fishes.

Gene duplication is important for the distribution of multiple functions among duplicated genes. Redundant gene copies can be classified into four categories: neofunctionalization, subfunctionalization, gene conservation back-up compensation, and dosage amplification (Kuzmin et al. 2022). Neofunctionalization is defined as one of the duplicated genes maintaining the full function of the ancestral gene and the other acquiring a new function; thus, both genes are retained. Subfunctionalization is defined as the function of an ancestral gene being partitioned among duplicated genes. In this model, duplicated genes are retained because both are necessary to maintain the function or expression pattern of the protein (Hughes 1994; Force et al. 1999; Stoltzfus 1999). Indeed, gene duplication has been implicated in the evolution of the vertebrate Aqp superfamily, with the *aqp2*, *aqp5*, and *aqp6* clusters showing different expression patterns in tetrapods as examples of subfunctionalization (Finn et al. 2014). As shown in this study, after tandem duplication, Aqp10.1 retained the full spectrum of plesiomorphic activities, whereas Aqp10.2 only retained water and glycerol permeabilities but lost urea and boric acid permeabilities. Although we do not have evidence for either of the two tandem duplicates acquiring a novel functionality, Aqp10.2 secondarily lost some of the ancestral subfunctions. Therefore, although we do not see a reciprocal loss of subfunctions for the Aqp10.1 paralog, the functional divergence of Aqp10 paralogs is most consistent with a subfunctionalization/functional specialization mechanism.

More specifically, what mechanism, then, could be responsible for the weakening of the urea and boric acid permeabilities of Aqp10.2? We propose two possibilities: 1) Aqp10.2 lost the structure necessary for urea and boric acid transport, or 2) Aqp10.2 acquired a filter that distinguishes urea and boric acid from glycerol and restricting urea and boric acid transport. In case 1), Aqp10.2 would be considered a type of subfunctionalization because it lost some functions of the ancestral gene and maintained some of them (fig. 6*B*) (He and Zhang 2005), and Aqp10.1 could potentially have lost some of the unknown functions of the ancestral gene. In case 2), Aqp10.2 retained some functions of the ancestral gene and gained a novel function, solute selectivity, and could be considered neofunctionalization (fig. 6*B*). Although the current data are more consistent with the subfunctionalization model due to loss of subfunctions for Aqp10.2, we cannot exclude the possibility of neofunctionalization by gain of novel solute selectivity in Aqp10.2 and hence functional specialization hidden as subfunction partitioning.

Structurally, water-specific Aqps have selective filters that block the permeability of molecules other than water (Hub and de Groot 2008; Gotfryd et al. 2018). The Asn-Pro- Ala (NPA) motif and the aromatic/Arg (ar/R) selectivity filter modulate the transport substrate specificity of Aqps. However, the mechanisms underlying solute selectivity among aquaglyceroporins have not been well studied. Kitchen et al. reported that human Aqp3 is highly permeable to glycerol but not to urea and that site-directed mutagenesis of human Aqp3, in which tyrosine 212 was mutated to alanine (Y212A), increases the urea permeability without affecting glycerol permeability (Kitchen et al. 2019). Therefore, Y212 is involved in the restriction of urea permeability in human Aqp3. However, Aqp10 of the African clawed frog, despite having a tyrosine residue at the same sites (supplementary fig. S2, Supplementary Material online), exhibits urea permeability (fig. 3). This result suggests that tyrosine residues may not solely contribute to the restriction of urea permeability in Aqp10. Kitchen et al. also stated that pore size is insufficient to explain the solute selectivity of Aqps and that the substrate discrimination in Aqps depends on a complex interplay between 1) the actual residues forming the ar/R region, 2) the physical size and chemical properties of the filter created by these residues, and 3) the structural context in which they are situated (Kitchen et al. 2019). Therefore, the question of how the Aqp10.2 of ray-finned fishes lost its urea and boric acid transport activity is critical. Because the solute selectivities of Aqp10, Aqp10.1, and Aqp10.2 have been characterized in this study, it is expected that a more detailed mechanism that influences the solute selectivity should and will be elucidated by future investigations. These future, mechanistic studies should lead to the determination of which model, subfunctionalization or neofunctionalization, is more consistent with the pleiotropy identified here.

## Materials and Methods

### Semiquantitative RT-PCR

Previously prepared total RNAs (Tran et al. 2006; Motoshima et al. 2023) were used in this study. First-strand complementary DNA was synthesized from 5 μg of total RNA using the SuperScript IV First-Strand Synthesis System (Thermo Fisher Scientific, Waltham, MA, USA) with oligo(dT) primers and analyzed by RT-PCR, as described previously (Tran et al. 2006; Motoshima et al. 2023). The cDNA was diluted 8-fold with nuclease-free water and used as a template for PCR with gene-specific primers (supplementary table S1, Supplementary Material online). Each reaction mixture (final volume, 12.5 μL) consisted of 0.25 μL cDNA (template), primers (individual final concentration, 0.25 μM), and 6.25 μL GoTaq Green Master Mix (2×; Promega, Madison, WI, USA).

The PCR conditions were as follows: initial denaturation at 94 °C for 2 min; 28 cycles (African clawed frog *aqp3*, *7*, *8*, *9*, *10*, and *actb*; spotted gar *aqp10s* and *actb*) of 94 °C for 15 s (denaturation); 52 °C (African clawed frog *aqp7*, *8*, *9*, *10*, and *actb*), 54 °C (African clawed frog *aqp3*), or 55 °C (spotted gar *aqp10s* and *actb*) for 30 s (annealing); 72 °C for 1 min (extension); and a final extension at 72 °C for 7 min. After amplification, 3 μL of the PCR mixture was diluted and loaded onto a microchip electrophoresis system for DNA/RNA analysis (MCE-202 MultiNA, Shimadzu, Kyoto, Japan) using a DNA-12000 reagent kit (Shimadzu) according to the manufacturer's instructions. Electrophoresis results were analyzed using the MultiNA Viewer software (Shimadzu).

### Cloning of Aqp10s from the African Clawed Frog, West African Lungfish, Gray Bichir, Spotted Gar, Zebrafish, and Pacific Herring

All animal protocols and procedures were conducted in accordance with the National Institutes of Health “Guide for the Care and Use of Laboratory Animals” and in accordance with a manual approved by the Institutional Animal Experiment Committee of the Tokyo Institute of Technology. Pacific herring were quickly decapitated, and intestinal tissues were dissected and stored in RNAlater solution (Thermo Fisher Scientific). We used Pacific herring but not Atlantic herring for cDNA cloning, because fresh tissues of Atlantic herring were not available in Japan. Total RNAs were isolated from the Pacific herring intestines using the acid guanidinium thiocyanate–phenol–chloroform extraction method with Isogen (Nippon Gene, Tokyo, Japan). The concentration and quality of the RNA were measured based on the UV absorbance at 260 and 280 nm and checked using the MultiNA and an RNA reagent kit (Shimadzu). First-strand complementary DNA was synthesized as described above and used to obtain full-length cDNAs of Aqp10s from the Pacific herring.

Full-length cDNAs of Aqp10s from the African clawed frog, spotted gar, and zebrafish were isolated from previously prepared intestinal cDNA of each species (Tran et al. 2006; Motoshima et al. 2023). Full-length cDNAs of Aqp10s from the West African lungfish and gray bichir Aqp10s were chemically synthesized (Eurofins Genomics, Tokyo, Japan).

cDNAs were amplified by PCR using a high-fidelity DNA polymerase (KOD-Plus-Neo or KOD One PCR Master Mix, Toyobo, Osaka, Japan), and primers were designed based on the genomic database of African clawed frog (*Xenopus laevis*, GCF_017654675.1) (Session et al. 2016), West African lungfish (*Protopterus annectens*, GCF_019279795.1) (Wang et al. 2021), Gray bichir (*Polypterus senegalus*, GCF_016835505.1) (Bi et al. 2021), Spotted gar (*Lepisosteus oculatus*, GCF_000242695.1) (Braasch et al. 2016), Zebrafish (*Danio rerio*, GCF_000002035.6) (Howe et al. 2013), Atlantic herring (*Clupea harengus*, GCF_900700415.2) (Martinez Barrio et al. 2016), (supplementary table S1, Supplementary Material online). cDNAs were subcloned into pGEMHE (Liman et al. 1992) with the In-Fusion Snap Assembly Master Mix (Takara Bio, Shiga, Japan) using gene-specific primers with 15 bp sequences complementary to the ends of the linearized pGEMHE (supplementary table S1, Supplementary Material online) and then sequenced. The newly identified sequences were deposited under their respective GenBank/EMBL/DDBJ accession numbers (spotted gar LocAqp10.1, LC767942, and LocAqp10.2, LC767946; Pacific herring CpaAqp10.1a, LC767943, CpaAqp10.2a, LC767944, and CpaAqp10.2b, LC767945). The sequences of African clawed frog XlaAqp10, West African lungfish PanAqp10, gray bichir PseAqp10.1 and 10.2, and zebrafish DreAqp10.1a and 10.2b were confirmed to be identical to those reported or predicted (XP_041429683.1, XP_043936577.1, XP_039607769.1, XP_039607747.1, NP_001002349, and XP_005159449, respectively).

### Expression of Aqp10s in *Xenopus* Oocytes

In addition to the expression vectors described above, we used previously prepared vectors for human Aqp10 (HsaAqp10) and Japanese pufferfish Aqp10.2b (TruAqp10.2b) as references (Ushio et al. 2022; Kumagai et al. 2023). The plasmids were linearized with NotI and capped RNAs (cRNAs) were transcribed in vitro using the T7 mMessage mMachine kit (Thermo Fisher Scientific). For PanAqp10, cRNA was transcribed in vitro using the T7 mMessage mMachine Ultra Kit (Thermo Fisher Scientific).

*Xenopus laevis* oocytes were dissociated with collagenase as described previously (Romero et al. 1998; Ushio et al. 2022; Kumagai et al. 2023) and injected with 50 nL of water or a solution containing 0.5 ng/nL cRNA (25 ng/oocyte), using a Nanoject II injector (Drummond Scientific, Broomall, PA, USA). Oocytes were incubated at 16 °C in OR3 medium and observed for 3–5 days after injection. The OR3 medium (1 L) consisted of 0.7% *w*/*v* powdered Leibovitz L-15 medium with L-glutamine (Thermo Fisher Scientific), 50 mL of 10,000 U penicillin, 10,000 U streptomycin solution in 0.9% NaCl (Sigma-Aldrich, St Louis, MO, USA), and 5 mM HEPES (pH 7.50). The osmolality was adjusted to 200 mOsmol/kg with NaCl powder (Romero et al. 1998; Ushio et al. 2022; Kumagai et al. 2023). The frogs were handled and the oocytes harvested according to the approved protocol described in the previous section.

### Swelling Assay

Oocyte swelling was monitored using a stereomicroscope (SZX9; Olympus, Tokyo, Japan) equipped with a charge-coupled device (CCD) camera (DS-Fi2; Nikon, Tokyo, Japan) as described previously (Ushio et al. 2022; Kumagai et al. 2023). The oocyte volumes were calculated assuming spherical geometry. Oocytes incubated with ND96 (∼200 mOsmol/kg) were transferred to 2-fold diluted ND96 (∼100 mOsmol/kg) for the water transport assays. For the glycerol, urea, and boric acid transport assays, oocytes were transferred to an isotonic solution containing ND96 supplemented with 180 mM glycerol, urea, or boric acid instead of NaCl and adjusted to an osmolality of ∼200 mOsmol/kg.

Water permeability (*P*_water_) was calculated from the osmotic swelling data and the molar volume of water (*V*_w_ = 18 cm^3^/mol) as follows (Preston et al. 1992): *P*_water_ = [*V*_o_ × *d*(*V*/*V*_o_)/*dt*]/[*S* × *V*_w_ × (osm_in_ – osm_out_)], where *S* is the initial oocyte surface area. Solute permeability (*P*_solute_) was calculated from the swelling data, total osmolality of the system (osm_total_ = 200 mOsmol/kg), and solute gradient (sol_out_ – sol_in_) as follows (Carbrey et al. 2003): *P*_solute_ = osm_total_ × [*V*_o_ × *d*(*V*/*V*_o_)/*dt*]/[*S* × (sol_out_ – sol_in_)].

The water, glycerol, urea, and boric acid transport activities of each Aqp10 were evaluated using oocytes from the same animal, and the experiment was repeated using a minimum of three frogs. Quantitative data are presented as mean ± standard deviation (SD) in table 1. *P*_water_ and *P*_solute_ values were compared among oocytes expressing Aqp10s and control oocytes, and the statistical significance for species with only one Aqp10 (human, African clawed frog, West African lungfish, and Japanese pufferfish) was evaluated using an unpaired *t*-test. For species with two or more Aqp10 paralogs, statistical significance was assessed using a one-way analysis of variance (ANOVA), followed by Tukey’s test, using GraphPad Prism software (version 5, GraphPad, San Diego, CA, USA). This software was also used to display the results in box plots.

## Supplementary Material

evad221_Supplementary_Data

## Data Availability

The data underlying this article are available in GenBank under accession numbers indicated in Materials and Methods.
