# Supplementary material for: Functional Divergence in Solute Permeability between Ray-Finned Fish-Specific Paralogs of aqp10
Source: Genome Biol Evol. 2023 Dec 1;16(1):evad221. doi: 10.1093/gbe/evad221 (PMC10769510; doi:10.1093/gbe/evad221)
Supplement: evad221_Supplementary_Data [file evad221_supplementary_data.zip › Supplementary Figures.pdf]

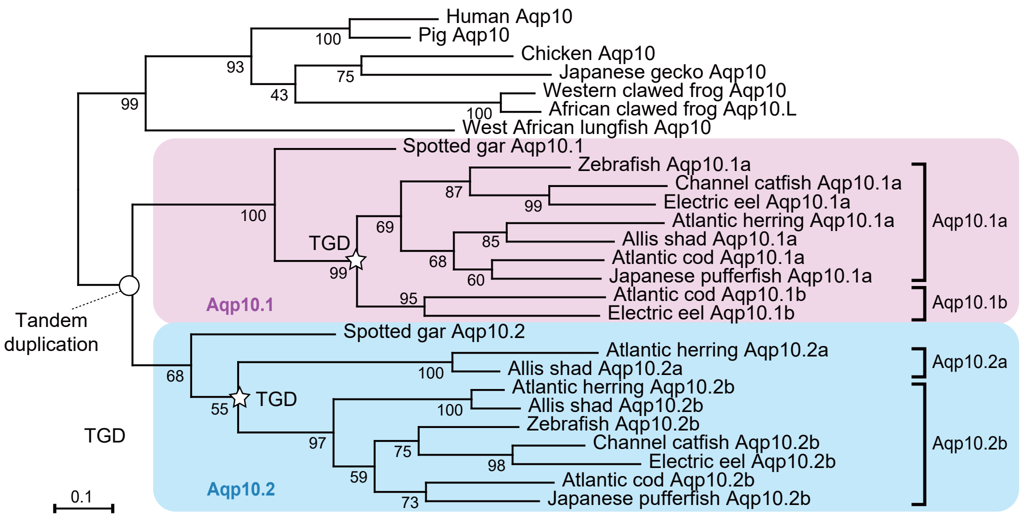

Supplementary Figure S1. Phylogenetic analyses of the amino acid sequences of Aqp10 in bony vertebrates. Vertebrate Aqp amino acid sequences were obtained from the following public databases: Ensembl genome browser (https://www.ensembl.org/) (Martin, et al. 2023), NCBI genome viewer (https://www.ncbi.nlm.nih.gov/genome/gdv/) (Rangwala, et al. 2021) and GenBank/EMBL/DDBJ (Supplementary Table S2). ClustalW (Chenna, et al. 2003) was used to align the amino acid sequences. The alignment composed of 27 sequences and 427 positions was used to construct a maximum-likelihood tree using the MEGA X software (Kumar, et al. 2018) with the JTT model (Jones, et al. 1992). An initial tree for the heuristic search was selected by the NJ/BioNJ method with the JTT model. The tree with the highest log likelihood (-11817.08) is shown. Bootstrap analyses were conducted with 100 bootstrap replicates (Felsenstein 1985).

Chenna R, et al. 2003. Multiple sequence alignment with the Clustal series of programs. Nucleic Acids Res 31: 3497-3500. doi: 10.1093/nar/gkg500

Felsenstein J 1985. Confidence Limits on Phylogenies: An Approach Using the Bootstrap. Evolution 39: 783-791. doi: 10.1111/j.1558-5646.1985.tb00420.x

Jones DT, Taylor WR, Thornton JM 1992. The rapid generation of mutation data matrices from protein sequences. Computer Applications in the Biosciences 8: 275-282. doi: 10.1093/bioinformatics/8.3.275

Kumar S, Stecher G, Li M, Knyaz C, Tamura K 2018. MEGA X: Molecular Evolutionary Genetics Analysis across Computing Platforms. Molecular Biology and Evolution 35: 1547-1549. doi: 10.1093/molbev/msy096

Martin FJ, et al. 2023. Ensembl 2023. Nucleic Acids Res 51: D933-D941. doi: 10.1093/nar/gkac958

Rangwala SH, et al. 2021. Accessing NCBI data using the NCBI Sequence Viewer and Genome Data Viewer (GDV). Genome Research 31: 159-169. doi: 10.1101/gr.266932.120

```
                                      1       10        20        30        40        50        60        70        80
HsaAqp10      ........................MVFTQAPAEIMGHLRIRSLLARQCLAEFLGVFVLMLLTQGAVAQAVTSGETKGNFFTMFLAGSLAVTIAIYVGGNVSGAHLN
XlaAqp10      MPRLYIRSRYLHPNSFFCIWRLSGMAWWRTRQDLRKWLRLRNPLARECLAEFLGVFVLILITVAATAQGVTSNETKGNFFCMYLAGAIAVVLAIHVSGGVSGGHLN
PanAqp10      .MLSYPPQTLVKFKHIPLKMPPAKSTMDKILLKAKRLIRIKSELGRHCLSEFFSTFMAMFFVQSSGAQLKTSYGEKGNYLSQTIGAALGITFSVYIAHGISGAHLN
PseAqp10.1    ..............................MQKLRHLLRLKNKLLKECLAEFLGTFVLLLFGCSAAAQVKTSHETKGQYLSVNMAFAMGVIFAVYISRGVSGAHLN
LocAqp10.1    ...................................................................................MSFAVGVMAAMYLCGGVSGAHLN
DreAqp10.1a   ..................................MKRMKVKNELARQIMGEILGTFVLLLFGCAAAAQVKTSRETKGQFLSGNIAFSVGVMSAMYLCRAVSGAHLN
CpaAqp10.1a   ..............................MKQVRKALTVKNELVRQCMGELLGTFVLLLFGCAAAAQVKTSRETKGQFLSANMAFSVGVMAAMYICKGVSGAHLN
PseAqp10.2    ..............................MEKVFKVLRVKNQLFKECLAELLGDYVLIIFGCGAVAQVTTSYDTKGQFLSINLAFAIGITFGVHIAHGISGAHLN
LocAqp10.2    ..............................MEKVQRLFQVQNQLLRQCLAELLGTYVMMMFGCGAVAQVTTSGEKKGDFLSINLAFALGVTFAVHVSGGVSGAHLN
TruAqp10.2b   ..............................MDGLLKNCRIKNQLLRECLAEFLGVYVLILFGCGSVAQVTTSHEKNGQYLSINLGFSLGVTFGVFVSRGVSGAHLN
DreAqp10.2b   ..............................MDRLLRRYRIKSRLPRECLAEFFGVYVLILFGCGSVAQVTTSQNTKGEYLSINLGFALGTTFGIYIAKGVSGAHLN
CpaAqp10.2a   ..............................MERARGLCINMKPLLRECLAEFLGVYIMILMGVGSMAQMITSKGTKGDYFSSTIAFALATTFAMYICGGVSGAQLN
CpaAqp10.2b   ..............................MDRLLRRCRIRSSLARECLAEGLGVYVMILFGCGSVAQVTTSRDTKGEYLSINLGFALGTTFGVYIAKGISGAHLN

                          90       100       110       120       130       140       150       160       170       180
HsaAqp10      PAFSLAMCIVGRLPWVKLPIYILVQLLSAFCASGATYVLYHDALQNYTGGNLTVTGPKETASIFATYPAPYLSLNNGFLDQVLGTGMLIVGLLAILDRRNKGVPAG
XlaAqp10      PAYSLSMCLLGRFPWWKLPLYTLIQLVGSFAGAAAAFALYYDAIQDYTKGNLTIFGPRETASIFCSYPAPYLSIANGFLDQVMGTAMLLVGILAIVDSKNKPVPQG
PanAqp10      PAYSLSMCLLRRLRWWKLPFYIIFQFLGSFLSAAAVYGLYYDAIQSYSGGNLTVTGPRETASIFFTLPASYLSIQNGFIDQVMGTAILIICLLAIGDPRNNPVPKG
PseAqp10.1    PAVSLSFCMIGRFPWTKVPIYIFFQIFGAYMASATVFALYYDAIMNYSGGNLTVTGSQETASIFATYPSDYLSHANAFFDQIVGTAVLLLCILPIEDAHNSPAPKG
LocAqp10.1    PAVSFSFCLLGRLQWRRLPFYSFSQILGAYVASAVVFLQYYDAILEFGQGSLTVTGPTETASIFATYPSEYLSLESGFFDQVVGTATLLLCILPLDDRLNSPAPKG
DreAqp10.1a   PAVSLSFCVLGDLAWIKLLPYSLAQILGAYLASGLVYLIYHDAIMEFSGGVLTVFGPNETASIFATYPTDVVSVQTNFLDQVVGTAMLMLCILPLNDKRNAPAPEA
CpaAqp10.1a   PAVSLSFCVLGQMSWKKLVPYSLSQLLGAYLASGLVFCVYYDAIMEFSGGVLTVTGPNETASIFATYPSEYVSLTSSFLDQIVGTAMLMMCILPLGDEHNTPAPPG
PseAqp10.2    PAVSLAFCLLGRFQWSKLPFYVLSQIIGAYLGAATVFAQYYDAIMSYGGGNLTVTGPTATAYIFATYPAAYLTTANGFVDQVVGTAALLLCLMALVDTRNSPAPKG
LocAqp10.2    PAVSLSLCVLGRHQWRRLPLYILSQVTGAFLAAATIYVVYYEAIMEYCGGNLTATGPNATAGIFATYPSSYLTLWNGFVDQVIGTGMLLLCVLALGDVNNSPAPPG
TruAqp10.2b   PAVSLSLCVLGRHPWKKLPFFILFQVFGAFLAAATVYLQYYDAIQTYSGGELTVTGPTATAGIFSTYPADYLSMWGGIVDQVIGTAALLLCVLALGDQRNTPLQDG
DreAqp10.2b   PAVSVSLCVLGRFSWTRLPFYVCSQLFGAFLAAATVALQYYDAIMDFTGGHLTVSGATATAGIFSTYPADYLSLWGGVVDQIIGTAALLVCVLALGDAHNTPAPAG
CpaAqp10.2a   PAVSFGMCVMGKHQWRKLPFYTLFQMLGSFCGAATVYALYYDAIMNLTQGKFTVTGPNATAHIFSTYPSGYLSIWNGFVDQVIGTAVLIVCSLALGDRRNSAAPTE
CpaAqp10.2b   PAVSLSLCLLGRHPWTHLPFYLVSQFLGAFLAAATVHLQYYDAISSFG..ALEVTGPNGTAGIFATYPADYLSLGAGVLDQVIGTAALLVCVLAIGDQRNTPAPQG

                   190       200       210       220       230       240       250       260       270       280       290
HsaAqp10      LEPVVVGMLILALGLSMGANCGIPLNPARDLGPRLFTYVAGWGPEVFSAGNGWWWVPVVAPLVGATVGTATYQLLVALHHPEGPEPAQ.DLVSAQHKASELETPAS
XlaAqp10      LEPIVVGMLVFSIGLSMGANCSYPINPTRDLGPRLFSAVAGWGLDVFRAGNNWWWVPVVAPCVGAVLGAILYQIFVEIHHPLAKDEEE.SPKEKEAQRGDMKEAQV
PanAqp10      LEPIAVGLLIICLGTTVGSNCGYAINPTRDIGPRIFTYLAGWGPEVFSAGNFWWWVPVVAPLVGSVLGTVLYQLFIGLQHPDLSDSKK.QENSPGAFILGNSVAES
PseAqp10.1    FEPVLVGFLVLGISMSMSSNCGAAINPARDLGPRLFTWSAGWGTAVFTAFENWWWIPIVAPMLGGVIGAYIYLLFIEFHHEDSDNMVQQSKSLTNQGDITSLANKG
LocAqp10.1    LVPPLVGLVVLGISMAMSSNCGAAINPARDLGPRLFTLTAGWGTEVFTAYRHWWWVPVVAPMLGSVLGSCLYLTFIQWHLPDPQGGPSPDAASRTKIQAPSAPAPH
DreAqp10.1a   LLPPIVATVVLGISISMSANCGAAINPARDLGPRLFTFTAGWGTEVFTCYDYFFWIPLVAPMVGGVLGSIIYLVFIQWHLPELEDESESGEMNDQTKVMEHNNKKD
CpaAqp10.1a   LIPPIVGAIVLGISTGMSSNCGGAINPARDLGPRLFTLTAGWGTEVFSCYGYWFWVPLVAPMIGGVIGTMVYQVFIQWHLPDSEAMEATDELVVTDLKDPSTKTSE
PseAqp10.2    LEPALVGMIVLVIGIAMGSNCGYAINPARDLGPRLFTYTAGWGLDVFTAGNYWCWVPLVAPLVGAILGCIIYLLFIEIHHSREEDEKQTNERGKTEYDIDLDEWKP
LocAqp10.2    LEPVLVGLVVLAIGISFGSNCGYPLNPARDLGPRLFTYIAGWGSEVFTAGGGWWWVPLLGPLVGALTGTALYQLLIELHHPGPGLGSGDTASGQAPPPEEAELGLP
TruAqp10.2b   VRPVLVGAAVLLIGISMGSNSGYAINPARDIGPRLFTYLMGWGAEVFTAGGGWWWVPIVAPCVGALLGTLVYELAIEVHHPPSAGELRTPSQGAPGTELELERDGK
DreAqp10.2b   LEPVLVGAAVLVIGISMGSNSGYAINPARDFGPRLFSYIAGWGDEVFRAGHGWWWVPIIVTCVGALLGSLLYELLIGVHHPDSEAVDHEDPTAALQQTVEMEGAQS
CpaAqp10.2a   LEPLMVGLVIWVIGLSMGSNCGYPLNPARDLGPRVFTYLAGWGSEVFSAGNGWFWVPLVAPFVGALVGAALYYFFIEIHHPPLEESANGDALCIGNKILKLEVPDQ
CpaAqp10.2b   LEPVLVGAIVLVIGVSMGSNSGYAINPARDLGPRVYTYLAGWGAEVFSAGGSWWWVPVVATPLGAVVGSLLYELLVGVHLPDSDTLADELILANHPALELEGKCPD

                  300
HsaAqp10      AQMLECKL......................................
XlaAqp10      FSISLDNPLSHRL.................................
PanAqp10      DLKKCQKEYNHKGADIIMGT..........................
PseAqp10.1    SKSGSFENIAVDGEMQMLRIDHDSKEEKPEERGYHISSRL......
LocAqp10.1    SDSETIAMPSWD..................................
DreAqp10.1a   EIYLKMSSI.....................................
CpaAqp10.1a   STIIKGTNL.....................................
PseAqp10.2    NKRNLTTLTASDKVHIINNSFQEGQSENSDWKNSSCNEECIVNRF.
LocAqp10.2    GEKGDERQDEGEACVTHRL...........................
TruAqp10.2b   AQ............................................
DreAqp10.2b   FDTIKENKKSGIFSITSADVG.........................
CpaAqp10.2a   DINALKSFKKDENLDNV.............................
CpaAqp10.2b   GEIPKPANPVNSQEWLERPTLGSAVCETAIKDAFPKCGGVHSLWGQ
```

Supplementary Figure S2. Multiple alignment of AQP10 protein sequences. The aromatic/Arg (ar/R) selectivity filters are shown in open magenta boxes. Conserved amino acids are shaded in black. The GenBank accession numbers are described in Supplementary Table S2. Multiple sequence alignment was performed using Clustal W and ESPript (Robert and Gouet 2014) was used to graphically display the results.

Robert X, Gouet P 2014. Deciphering key features in protein structures with the new ENDscript server. Nucleic Acids Res 42: W320-324. doi: 10.1093/nar/gku316
