# Supplementary material for: Functional Divergence in Solute Permeability between Ray-Finned Fish-Specific Paralogs of aqp10
Source: Genome Biol Evol. 2023 Dec 1;16(1):evad221. doi: 10.1093/gbe/evad221 (PMC10769510; doi:10.1093/gbe/evad221)
Supplement: evad221_Supplementary_Data [file evad221_supplementary_data.zip › Supplementary Tables.pdf]

Supplementary Table S1. List of primers used for polymerase chain reaction amplification of Aqp10s in bony vertebrates

Species	Gene	Accession	Remarks	Direction	Sequence (5' to 3')
African clawed frog	*aqp10.L*	XM_041573749.1	RT-PCR	Fw	GTCACCTGAACCCTGCCTAC
				Rv	ACGCTGTCTCTCTAGGTCCA
			Full-length cDNA	Fw	ATGCCCAGGCTTTATATAAGGAGCA
				Rv	TCACAGGCGGTGGGAGAG
			In-Fusion cloning	Fw	gcagatcattccccATGCTAGGAATGCCTAGACTTTATAT
				Rv	agaattcggatccccTCACAGACGGTGGGAGAGGGGATT
Spotted gar	*aqp10.1*	LC767942	RT-PCR	Fw	GCTGGCGATTCGGAACCA
				Rv	CTGTTGAGCCTGTCGTCCAA
			Full-length cDNA	Fw	ATGGAGAAGGTGAAGCGCGTGCTG
				Rv	*TCAGGGGTCCCAGCTGGGCATGG*
			In-Fusion cloning	Fw	gcagatcaattccccATGGAGAAGGTGAAGCGCG
				Rv	agaattcggatccccTCAGGGGTCCCAGCTGG
Spotted gar	*aqp10.2*	XM_015368732.1<br>LC767946	RT-PCR	Fw	ACAGAGACTGTTCCAGGTACA
				Rv	CATCACCCAGAGCCAGAACA
			Full-length cDNA	Fw	ATGGAGAAGGTACAGAGACTGTTCCAGGTACAGAA
				Rv	ATAGAGCTCTTCCCTGTGGCAGGTGTTTCCCGTTG
			In-Fusion cloning	Fw	gcagatcaattccccaATGGAGAAGGTACAGAGACTG
				Rv	agaattcggatccccTCAGAGGCGGTGGGTGAC
Zebrafish	*aqp10.1a*	NM_001002349.1	Full-length cDNA	Fw	ATGAAGAGGATGAAGGTGAAAAATGAACTGGCACG
				Rv	ttccagactttTTAAATTGAAGACATTTTAAGG
			In-Fusion cloning	Fw	gcagatcaattccccaATGAAGAGGATGAAGGTGAAAAATG
				Rv	agaattcggatccccTTAAATTGAAGACATTTTAAGG
Zebrafish	*aqp10.2b*	XM_005159392.4	Full-length cDNA	Fw	ATGGACCGTCTGCTGAGGA
				Rv	TTACCCTACGTCTGCTGAGGTTAT
			In-Fusion cloning	Fw	gcagatcattccccATGGACCGTCTGCTGAGGAGATAC
				Rv	agaattcggatccccTTACCCTACGTCTGCTGAGGTTATAG
Pacific herring	*aqp10.1a*	LC767943	Full-length cDNA	Fw	TTGACCGGCAGAATGAAGCAGGTGAG
				Rv	CTATAGGTTCGTTCCTTTAATGATTGTCG
			In-Fusion cloning	Fw	gcagatcattccccTTGACCGGCAGAATGAAGC
				Rv	agaattcggatccccCTATAGGTTCGTTCCTTTAA
Pacific herring	*aqp10.2a*	LC767944	Full-length cDNA	Fw	ATGGAAAGAGCACGAGGACTATGC
				Rv	TCACACATTGTCCAAATTCTCATCC
			In-Fusion cloning	Fw	gcagatcattccccATGGAAAGAGCACGAGGAC
				Rv	agaattcggatccccTCACACATTGTCCAAATTC
Pacific herring	*aqp10.2b*	LC767945	Full-length cDNA	Fw	ATGGATCGGCTTTTAAGGAGGTGTC
				Rv	TCATTGCCCCCAGAGGCTGTGCACT
			In-Fusion cloning	Fw	gcagatcattccccATGGATCGGCTTTTAAGGAG
				Rv	agaattcggatccccTCATTGCCCCCAGAGGCTG
West African lungfish	*aqp10*	XM_044080642.1	In-Fusion cloning	Fw	gcagatcattccccATGCTTTCATACCCACCACA
				Rv	agaattcggatccccCTAAGTACCCATTATTATGT
Gray bichir	*aqp10.1*	XM_039751835.1	In-Fusion cloning	Fw	gcagatcattccccATGCAGAAACTAAGACATC
				Rv	agaattcggatccccTTAAAGCCGGCTACTGATG
Gray bichir	*aqp10.2*	XM_039751813.1	In-Fusion cloning	Fw	gcagatcattccccATGGAGAAAGTGTTTAAAG
				Rv	agaattcggatccccTTAAAACCTATTGACAATAC
West African lungfish	*aqp10*	XM_044080642.1	In-Fusion cloning	Fw	gcagatcattccccATGCTTTCATACCCACCACA
				Rv	agaattcggatccccCTAAGTACCCATTATTATGT
African clawed frog	*aqp7.L*	XM_018226930.2	RT-PCR	Fw	CTGGATACGCCATCAACCCAG
				Rv	CCACTTTGGTCTCTTCTTCCTCC
African clawed frog	*aqp8.L*	XM_018237062.2	RT-PCR	Fw	AAATCATGCCCCACTGGTTTGAC
				Rv	CAGCAGGATTAAAATGGCCACC
African clawed frog	*aqp9.L*	XM_018253027.2	RT-PCR	Fw	GGGATGTGCAATGAATCCTGC
				Rv	GGGTATCTTCCATGTCATTGCTG
African clawed frog	*actb.L*	NM_001088953.2	RT-PCR	Fw	GACAGTCTGTGTGCGTCCAA
				Rv	TGGGCGACCCACAATAGATG
Spotted gar	*actb*	XM_006637121.2	RT-PCR	Fw	TGCCGCACTGGTTGTTGATA
				Rv	GAAGCTGTAGCCTCTCTCGG

Supplementary Table S2. Amino acid sequences of Aqp10s used for the phylogenetic analysis.

Gene	Species	Accession number
aqp10	Human (*Homo sapiens*)	NP_536354
aqp10	Pig (*Sus scrofa*)	NP_001121926
aqp10	Chicken (*Gallus gallus domesticus*)	XP_015154084
aqp10	Japanese gecko (*Gekko japonicus*)	XP_015274582.1
aqp10	African clawed frog (*Xenopus laevis*)	XP_041429683.1
aqp10	Western clawed frog (*Xenopus tropicalis*)	XP_017945847
aqp10	West African lungfish (*Protopterus annectens*)	XP_043936577.1
aqp10.1	spotted gar (*Lepisosteus oculatus*)	LC767942
aqp10.2	spotted gar (*Lepisosteus oculatus*)	LC767946
aqp10.1a	zebrafish (*Danio rerio*)	NP_001002349
aqp10.2b	zebrafish (*Danio rerio*)	XP_005159449
aqp10.1a	channel catfish (*Ictalurus punctatus*)	ENSIPUT00000006294.1
aqp10.2b	channel catfish (*Ictalurus punctatus*)	ENSIPUT00000011195.1
aqp10.1a	allis shad (*Alosa alosa*)	XM_048261394
aqp10.2a	allis shad (*Alosa alosa*)	XM_048261395
aqp10.2b	allis shad (*Alosa alosa*)	XM_048229129
aqp10.1a	Atlantic herring (*Clupea harengus*)	ENSCHAT00000039518.1
aqp10.2a	Atlantic herring (*Clupea harengus*)	ENSCHAT00000037340.1
aqp10.2b	Atlantic herring (*Clupea harengus*)	ENSCHAT00000034239.1
aqp10.1a	Atlantic cod (*Gadus morhua*)	ENSGMOT00000031448.1
aqp10.1b	Atlantic cod (*Gadus morhua*)	ENSGMOT00000011855.2
aqp10.2b	Atlantic cod (*Gadus morhua*)	ENSGMOT00000045998.1
aqp10.1a	electric eel (*Electrophorus electricus*)	ENSEEET00000015527.1
aqp10.1b	electric eel (*Electrophorus electricus*)	ENSEEET00000013246.1
aqp10.2b	electric eel (*Electrophorus electricus*)	ENSEEET00000013398.1
aqp10.1a	Japanese pufferfish (*Takifugu rubripes*)	LC735291
aqp10.2b	Japanese pufferfish (*Takifugu rubripes*)	LC735292

Supplementary Table S3. Genome databases used for the synteny analysis.

Species	Genome database	Reference
Human (*Homo sapiens*)	GCF_000001405.40	(Lander, et al. 2001)
Mouse (*Mus musculus*)	GCF_000001635.27	(Mouse Genome Sequencing, et al. 2002)
Pig (*Sus scrofa*)	GCF_000003025.6	(Groenen, et al. 2012)
Chicken (*Gallus gallus domesticus*))	GCF_016699485.2	(International Chicken Genome Sequencing 2004)
Japanese gecko (*Gekko japonicus*)	GCF_001447785.1	(Liu, et al. 2015)
African clawed frog (*Xenopus laevis*)	GCF_017654675.1	(Session, et al. 2016)
Coelacanth (*Latimeria chalumnae*)	GCF_000225785.1	(Amemiya, et al. 2013)
West African lungfish (*Protopterus annectens*)	GCF_019279795.1	(Wang, et al. 2021)
Gray bichir (*Polypterus senegalus*)	GCF_016835505.1	(Bi, et al. 2021)
Spotted gar (*Lepisosteus oculatus*)	GCF_000242695.1	(Braasch, et al. 2016)
Atlantic herring (*Clupea harengus*)	GCF_900700415.2	(Martinez Barrio, et al. 2016)
Allis shad (*Alosa alosa*)	GCF_017589495.1	(Sabatino, et al. 2022)
Zebrafish (*Danio rerio*)	GCF_000002035.6	(Howe, et al. 2013)
Channel catfish (*Ictalurus punctatus*)	GCF_001660625.3	(Chen, et al. 2016)
Electric eel (*Electrophorus electricus*)	GCF_013358815.1	(Gallant, et al. 2014)
Atlantic cod (*Gadus morhua*)	GCF_902167405.1	(Star, et al. 2011)
Japanese medaka (*Oryzias latipes*)	GCF_002234675.1	(Kasahara, et al. 2007)
Nile tilapia (*Oreochromis niloticus*)	GCF_001858045.2	(Brawand, et al. 2014)
Japanese pufferfish (*Takifugu rubripes*)	GCA_901000725.3	(Aparicio, et al. 2002)

Amemiya CT, et al. 2013. The African coelacanth genome provides insights into tetrapod evolution. Nature 496: 311-316. doi: 10.1038/nature12027

Aparicio S, et al. 2002. Whole-genome shotgun assembly and analysis of the genome of *Fugu rubripes*. Science 297: 1301-1310.

Bi X, et al. 2021. Tracing the genetic footprints of vertebrate landing in non-teleost ray-finned fishes. Cell 184: 1377-1391 e1314. doi: 10.1016/j.cell.2021.01.046

Braasch I, et al. 2016. The spotted gar genome illuminates vertebrate evolution and facilitates human-teleost comparisons. Nature Genetics 48: 427-437. doi: 10.1038/ng.3526

Brawand D, et al. 2014. The genomic substrate for adaptive radiation in African cichlid fish. Nature 513: 375-381. doi: 10.1038/nature13726

Chen X, et al. 2016. High-quality genome assembly of channel catfish, *Ictalurus punctatus*. Gigascience 5: 39. doi: 10.1186/s13742-016-0142-5

Gallant JR, et al. 2014. Nonhuman genetics. Genomic basis for the convergent evolution of electric organs. Science 344: 1522-1525. doi: 10.1126/science.1254432

Groenen MA, et al. 2012. Analyses of pig genomes provide insight into porcine demography and evolution. Nature 491: 393-398. doi: 10.1038/nature11622

Howe K, et al. 2013. The zebrafish reference genome sequence and its relationship to the human genome. Nature 496: 498-503. doi: 10.1038/nature12111

International Chicken Genome Sequencing C 2004. Sequence and comparative analysis of the chicken genome provide unique perspectives on vertebrate evolution. Nature 432: 695-716. doi: 10.1038/nature03154

Kasahara M, et al. 2007. The medaka draft genome and insights into vertebrate genome evolution. Nature 447: 714-719. doi: 10.1038/nature05846

Lander ES, et al. 2001. Initial sequencing and analysis of the human genome. Nature 409: 860-921. doi: 10.1038/35057062

Liu Y, et al. 2015. *Gekko japonicus* genome reveals evolution of adhesive toe pads and tail regeneration. Nat Commun 6: 10033. doi: 10.1038/ncomms10033

Martinez Barrio A, et al. 2016. The genetic basis for ecological adaptation of the Atlantic herring revealed by genome sequencing. Elife 5. doi: 10.7554/eLife.12081

Mouse Genome Sequencing C, et al. 2002. Initial sequencing and comparative analysis of the mouse genome. Nature 420: 520-562. doi: 10.1038/nature01262

Sabatino SJ, et al. 2022. The genetics of adaptation in freshwater Eurasian shad (Alosa). Ecol Evol 12: e8908. doi: 10.1002/ece3.8908

Session AM, et al. 2016. Genome evolution in the allotetraploid frog *Xenopus laevis*. Nature 538: 336-343. doi: 10.1038/nature19840

Star B, et al. 2011. The genome sequence of Atlantic cod reveals a unique immune system. Nature 477: 207-210. doi: 10.1038/nature10342

Wang K, et al. 2021. African lungfish genome sheds light on the vertebrate water-to-land transition. Cell 184: 1362-1376 e1318. doi: 10.1016/j.cell.2021.01.047
